# Carbonized Micro- and Nanostructures: Can Downsizing Really Help?

**DOI:** 10.3390/ma7053820

**Published:** 2014-05-14

**Authors:** Mohammad Naraghi, Sneha Chawla

**Affiliations:** 1 Department of Aerospace Engineering, Texas A and M University, 3409 TAMU College Station, TX 77843-3409, USA; 2 Department of Material Science and Engineering, Texas A and M University, 3003 TAMU College Station, TX 77843-3003, USA; E-Mail: sachawla@gmail.com

**Keywords:** carbon nanofibers, carbon fibers, turbostratic domains, skin–core inhomogeneity

## Abstract

In this manuscript, we discuss relationships between morphology and mechanical strength of carbonized structures, obtained via pyrolysis of polymeric precursors, across multiple length scales, from carbon fibers (CFs) with diameters of 5–10 μm to submicron thick carbon nanofibers (CNFs). Our research points to radial inhomogeneity, skin–core structure, as a size-dependent feature of polyacrylonitrile-based CFs. This inhomogeneity is a surface effect, caused by suppressed diffusion of oxygen and stabilization byproducts during stabilization through skin. Hence, reducing the precursor diameters from tens of microns to submicron appears as an effective strategy to develop homogeneous carbonized structures. Our research establishes the significance of this downsizing in developing lightweight structural materials by comparing intrinsic strength of radially inhomogeneous CFs with that of radially homogeneous CNF. While experimental studies on the strength of CNFs have targeted randomly oriented turbostratic domains, via continuum modeling, we have estimated that strength of CNFs can reach 14 GPa, when the basal planes of graphitic domains are parallel to nanofiber axis. The CNFs in our model are treated as composites of amorphous carbon (matrix), reinforced with turbostratic domains, and their strength is predicted using Tsai–Hill criterion. The model was calibrated with existing experimental data.

## 1. Introduction

Different forms of graphitic carbon, both synthetic and natural, with length scales spanning from a few microns to nanometers—such as carbon/graphite fibers, carbon nanotubes and nanofibers, or graphite nanoparticles—have been at the heart of significant industrial developments and scientific research in the past few decades [1–6]. These efforts are largely driven by the remarkable physical properties of these structures, such as their mechanical strength which routinely exceeds ~4–5 GPa, and can be as high as ~100 GPa, especially in the nanoscale [1–3,5,7]. Apart from excellent mechanical performance, graphitic structures are known to have remarkable electrical conductivity [8,9] and thermal stability [10,11], partly owed to the delocalized electrons in the graphitic domains and the strong inplane covalent bonds between carbon atoms.

As structural materials, the remarkable mechanical properties of graphitic carbon have led to significant breakthroughs in many areas such as aviation industry and space missions. In this regard, one of the most inspiring stories of success is the airliner Boeing 787, which benefits from carbon fiber composites as the most abundantly used material in the structural components [12]), mostly due to its exceptional specific strength [13]. For a comprehensive list of carbon fiber applications, interested readers may refer to [1].

Carbon fibers (CFs) are fabricated via thermal stabilization and carbonization of polymeric microfibers, which are dominantly polyacrylonitrile (PAN)-based [1,13]. In the past decade or so, the technology of carbon fibers has been applied to polymeric nanofibers, to develop carbon nanofibers (CNFs) [3,4,14,15]. The microstructure of individual CNFs is composed of amorphous carbon, turbostratic domains and graphitic regions, with relative concentrations that can be controlled via processing parameters, such as carbonization temperature [3,15]. From a mechanical standpoint, CFs and CNFs are very stiff structures, with moduli that is tunable within 100–900 GPa [1,3,15,16]. The upper bound is materialized at relatively high carbonization temperatures (typically exceeding 2000 °C), where substantial graphitization has taken place. Therefore, it is not surprising that this upper bound is comparable to moduli of graphitic nanostructure, such as CNTs and graphene [1,2,5,7,17]. This is despite the significant characteristic length scale differences between CNTs and carbon fibers.

While the modulus of graphitic materials is only marginally affected by length scale, the experimentally realized strength of typical carbon fibers and nanofibers is in the range of ~1–8 GPa [1,3,15,18], which is an order of magnitude lower than the experimentally measured strength of graphitic nanostructures such as CNTs and graphene [5,7]. This relatively low strength of CFs and CNFs is potentially due to a combination of factors such as radial inhomogeneities in CFs, misorientation of graphitic layers with respect to the nanofiber axis in typical CNFs, and structural defects such as microcracks and holes in the material [1,18,19].

Despite the significantly more appealing strength of CNTs, their industrial scale application is still pending upon the development of scalable manufacturing techniques which are capable of producing low defect density CNTs. This is in contrast to CFs which are produced on industrial scales [13]. Similarly, given the scalability of the production of electrospun PAN nanofibers, the production of electrospun CNFs in large quantities are imaginable, while better control of the processing parameters are required to enhance their mechanical properties [20].

In this manuscript, we focus on the microstructure of carbonized structures, mainly CFs with diameters of ~5–10 μm and CNFs with submicron diameters, with an emphasis on establishing relationships between microstructure and mechanical properties of the two. Our discussions will be largely limited to polyacrylonitrile (PAN)-based CFs and CNFs, as PAN is the most abundant precursor of carbonized structures [3,13,15,20,21]. That is mostly due to the high carbon yield of PAN (theoretically as high as 67 wt%) and its relatively modest stabilization temperatures (below 300 °C), leading to decomposition prior to melting [1,20]. More specifically, we will demonstrate the potentials of downsizing the technology of carbon fibers to submicron diameter filaments, to achieve significantly higher strength compared to carbon fibers. For this purpose, we will first describe the mechanical properties and microstructure of CFs, in light of their inherent radial inhomogeneities, and the consequent flaws. We will then point to the size-dependency of the radial inhomogeneity in CFs, according to which, sufficiently thin carbonized filaments with submicron diameters (CNFs), are expected to be radially homogeneous. To conclude, we will then estimate the theoretical strength of such homogeneous CNFs as a function of the graphitic content and graphitic domain alignment.

## 2. Carbonized Structures: Fabrication and Microstructure

Typical carbon/graphite fibers (CFs) with diameters of a few microns to as thick as ~10 μm, are fabricated via thermal stabilization and carbonization of polymeric microfibers, also known as the precursor [1]. The most common type of precursors for carbon fibers are polyacrylonitrile (PAN) homopolymers and copolymers [1,13,20]. Thermal stabilization is an exothermic process that takes place in the presence of oxygen (typically in air), during which PAN precursors will lose hydrogen (dehydrogenation), and partly transform into cyclic “ladder-like” structures (cyclization) [13,22]. Oxygen is a prerequisite for proper thermal stabilization, as it facilitates both the dehydrogenation and the initiation of the cyclization processes [23]. Subsequently, stabilized PAN is carbonized in inert atmosphere at temperatures of 800 °C to 2000 °C. The carbonized structure is composed of turbostratic/graphitic domains and amorphous carbon. The carbonized nanofibers may also be heated to higher temperatures to enhance the degree of graphitization [13,20]. In this process, it is essential to develop molecular alignment in PAN precursors via, for instance, hot drawing, as a means to enhance graphitic orientational order in CFs and leading to stronger and stiffer CFs [24,25]. More details of the fabrication are in [13].

While the production of carbon fibers with strength of over 3 GPa is at least half a century old [1,20,26], in the past decade or so, this technology branched out into developing nanoscale carbon fibers (CNFs). The production of CNFs has become possible specifically after the reemergence of electrospinning, as a scalable method to produce precursor nanofibers, as individual and bundles of filaments [3,4,15,16,27]. The most common precursor for CNFs is PAN, which is electrospun from a solution of PAN in dimethylformamide (DMF) [3,15,16], although other precursors have also been used [4]. Adopted from the carbon fiber industry, the transformation of electrospun polymer nanofibers into CNFs is carried out through thermal stabilization and carbonization of precursors [3,4,15,16,27].

Despite the similarities between their precursor types and thermal treatments in the fabrication process, the microstructure of the current state of the art CFs and CNFs are markedly different. On one hand, the microstructure of PAN-based carbon fibers shows a distinct radial inhomogeneity, known as skin–core structure [28,29]. The skin contains graphitic/turbostratic domains, with a high degree of alignment (their basal plane is more or less parallel to the fiber axis), while the core contains less oriented and more interwoven turbostratic domains. Given the fact that CFs are developed at very high temperatures (>800 °C), this radial inhomogeneity promotes the formation of voids and microcracks, many of which are formed during cooling to room temperature, due to a mismatch between the thermal expansion coefficients of the skin and the core [30]. In contrast to CFs, the processing parameters of CNF microstructure can be tuned such that turbostratic domains are homogeneously dispersed within the nanofiber [3]. Moreover, unlike the graphitic alignment in the skin of CFs, the turbostratic domains in CNFs are nearly randomly oriented [3,15,21]. Considerations of these differences between CFs and CNFs may open new horizons in developing carbonized structures with exceptional mechanical performance that will surpass the strength of CFs.

### 2.1. Origin of Radial Inhomogeneity in Carbonized Structures

It is generally accepted that the skin–core structure in PAN-based CFs is rooted in the thermochemical reactions that take place during thermal stabilization [18,23,25,26,31]. The dehydrogenation and cyclization processes during thermal stabilization of PAN are facilitated by the presence of oxygen [23]. The required oxygen is supplied by the environment, via a diffusion process, through the fiber skin. In addition, during the stabilization process, volatile species and heat are generated (thermal stabilization is an exothermic process), which are to be dissipated, via diffusion from the fiber core to the skin, and from the skin to the environment [3]. Therefore, as pointed out by Liu *et al.* [26], the stabilization process is highly controlled by the inward rate of diffusion of oxygen and the outward diffusion rate of byproducts of PAN stabilization. Hence, it is expected that the concentration of oxygen will be highest on the precursor skin. As such, it seems that the fiber skin may become fully stabilized, due to its proximity with the environment (source of oxygen), while the core may get only partially stabilized due to insufficient oxygen. Moreover, stabilization will increase the concentration of the stabilization byproducts at the core, which may slow down the rate of stabilization of the core. Another factor which may adversely affect the quality of stabilized PAN is the heat generated during the exothermic stabilization process. Since the heat can be dissipated to the environment through the skin, the local rise in the temperature, and thus thermal degradation, is expected to be higher at the core. However, more evidence is required to establish the magnitude and significance of the local rise in temperature.

The microstructural differences between the core and the skin can readily be identified via X-ray and electron diffraction techniques. However, direct observation of the cross-section of CFs with high resolution microscopy techniques, such as scanning electron or optical microscopy, will not reveal a distinct skin–core structure, except in extreme cases of a poorly stabilized core [18,29]. For instance, during carbonization, a poorly stabilized PAN core may melt and conform to the stabilized PAN, leaving a hollow core inside the developing CF [26,32].

### 2.2. Characteristic “Thickness” of Fully Stabilized Skin

As pointed out in the previous sections, the formation of the skin is primarily a surface effect, which is highly controlled by the diffusion of oxygen to the fiber and byproducts out of the fiber through the surface. Therefore, the thickness of the skin is expected to be independent of the fiber diameter. The skin in this context refers to the outmost layer of a carbonized structure with little or no variations in the quality of the carbonized structures (for instance, the G to D peak in Raman spectra). As such, reducing the diameter of precursors of CFs should reduce the thickness of the unstabilized or insufficiently stabilized core. Moreover, sufficiently thin CFs should be free from this inhomogeneity. The critical diameter associated with the “sufficiently thin CFs” can be estimated as the thickness of the skin in typical CFs, which is ~1–2 μm, that contain a high degree of graphitic alignment [18,32]. Similarly, CNFs with diameters of ~1 μm or less, in general, do not show radial inhomogeneity, supporting the hypothesis that the radial inhomogeneity is controlled by diffusion with a critical depth of ~1 μm [3].

**Figure 1.**
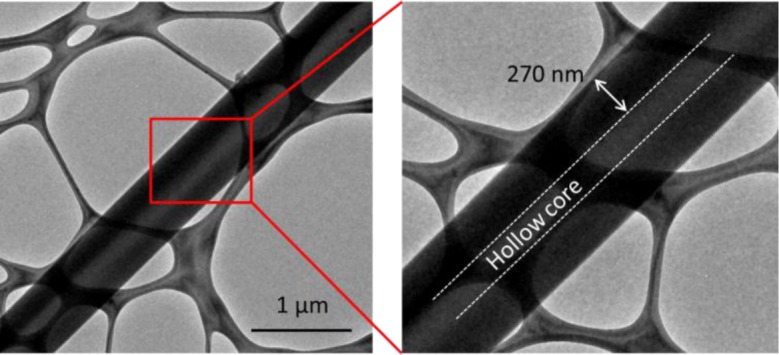
TEM image of electrospun CNFs, fabricated through accelerated stabilization of electrospun PAN nanofibers, at thermal stabilization temperatures of 294 °C.

However, given its diffusive nature, the thermal stabilization of CFs is not only controlled by the penetration depth of oxygen, but also by the rate of the stabilization of the core. That is, under accelerated stabilization conditions, e.g., relatively high stabilization temperatures, CNFs with radial inhomogeneity may also form. Moreover, rapid thermal stabilization may also induce radial inhomogeneity in submicron carbonized fibers. To further demonstrate this effect, we fabricated two types of CNFs (type I and II) by electrospinning a 12 wt% solution of PAN in DMF at a respective electrospinning distance and voltage of 20 cm and 16 kVs. CNFs of types I and II were fabricated by thermal stabilization of PAN nanofibers at 264 °C and 294 °C, respectively, followed by carbonization at 1100 °C. SEM images revealed that the diameter of the nanofibers were mostly in the range of 200–800 nm. The samples were then observed in TEM. The type I CNFs (stabilized at 264 °C) did not show any sign of radial inhomogeneity. However, in type II (stabilized at 294 °C), there seems to be a critical diameter of ~500 nm above which nanofibers with a hollow core will form, as shown in Figure 1.

The formation of the hollow cores in CNFs can be attributed to the poorly stabilized/unstabilized PAN core, which melts during carbonization and conforms to the surrounding PAN, a mechanism proposed by Liu *et al*. [26]. The poor stabilization of the core, as pointed out in the previous section, is a result of the low rate of oxygen and stabilization byproduct diffusion through the skin. In addition, the formation of hollow cores in type II, in contrast to solid (filled) CNFs of type I, can be taken as an indication that the thermal stabilization of the PAN suppresses oxygen diffusivity. In other words, higher thermal stabilization temperature in type II could have led to accelerated thermal stabilization of PAN skin, preventing or suppressing further diffusion of oxygen to the core. This is in contrast to case I, in which slow thermal stabilization of the skin could only marginally affect the diffusion of oxygen to the core. As such, in this case, the maximum penetration depth of oxygen from the surface of PAN can be approximated to be equal to the thickness of the solid skin of CNFs, as presented in Figure 1 and Table 1, which is ~250–400 nm.

Therefore, there seem to be two parameters controlling the formation of radial inhomogeneity in carbonized structures: the thickness of the sample and the rate of thermal stabilization. While generally samples with thicknesses comparable or less than the penetration depth of oxygen diffusion tend to be uniform, rapid stabilization of the skin may suppress the skin’s oxygen diffusivity, lowering the oxygen content reaching the core. Depending on the temperature of the stabilization, the oxygen penetration depth may vary from ~0.5 μm to 2 μm, with lower stabilization temperatures favoring the lower bound.

### 2.3. Radial Inhomogeneity Affects the Mechanics of Carbonized Structures

As mentioned in the introduction, the modulus of carbon fibers compares favorably with the corresponding value of graphene, while the strength of the former is only a small fraction of the latter. This is despite the fact that carbon fibers are mostly composed of layers of sp^2^ hybridized carbon atoms, similar to graphene [32].

One hypothesis for this poor comparison of the strength is the classical size effect in brittle materials. In other words, according to this hypothesis, carbon fibers are likely to be weaker than graphene nanoparticles and CNTs, simply because of their larger characteristic lengths and the consequent higher possibility of existing defects with critical size in them compared to nanomaterials. Defects in CFs include atomic scale defects such as imperfect stacking between graphene layers and dislocations, mesoscale defects such as undulation of graphene ribbons, and microscale defects such as pores [1,32]. The latter may form during the processing steps of the precursor, such as drawing [33]. The significance of classical size effect can be evaluated by estimating the intrinsic strength of CFs. Similar to other brittle materials, the intrinsic strength of CFs can be estimated as the hypothetical strength of CFs at zero length, by extrapolating the strength of CFs as a function of gage length, for instance, with the aid of a Weibull distribution of tensile strength [1,34]. However, even the intrinsic strength of CFs (at zero length), 6–10 GPa [1,34], is a small fraction of the strength of graphene sheets [5,7]. Therefore, a classical size effect does not seem to be sufficient to explain the significantly lower strength of CFs compared to graphene sheets and CNTs.

On the other hand, the microstructure of PAN-based CFs points to the development of other types of flaws, specific to CFs, which may reduce the intrinsic strength of CFs to values significantly lower than CNTs or graphene. In this regard, one needs to consider the skin–core structure of CFs at room temperature, forming at temperatures higher than 800 °C, when the radially inhomogeneous stabilized PAN is being carbonized. In other words, the thermal contraction of the radially inhomogeneous structure of CFs will develop residual stresses in multiple locations across the fiber. One source of the thermal residual stress is the anisotropic thermal contraction of sp^2^ hybridized carbon atom regions (graphene domains), which leads to cleavage cracks along the c-plane [32]. In addition, if domains are misoriented with respect to each other, the free thermal contraction strains of each domain will be different from its neighboring domains, due to anisotropic thermal contraction of graphene. Therefore, thermal stresses are expected to increase with the order of misorientation of neighboring graphitic domains. Hence, the CF core that contains more randomly oriented domains is expected to contain more residual thermal stresses.

Another source of thermal residual stresses is the inhomogeneous thermal contraction of CFs between the fiber core and the skin, which leads to the formation of microcracks between the core and the skin [32] and may lower the intrinsic strength of the carbon fibers [28].

### 2.4. Mechanics of CNFs and Carbon Fibers in Retrospect

The structural performance of carbon fibers is inherently compromised by their skin–core structure, and the consequent residual thermal stresses and voids between the core and the skin [13,20,28,29]. On the contrary, the sub-micron diameter of electrospun PAN nanofibers facilitates the formation of homogeneous electrospun CNFs as observed by Naraghi and his colleagues [3], potentially due to enhanced diffusion of oxygen and stabilization byproducts to the nanofiber core across its significantly thinner cross section compared to carbon fibers. This is consistent with the fact that the typical thickness of the skin in carbon fibers, with aligned graphitic domains, is several hundred nanometers, comparable to the diameter of individual CNFs [3,18,29].

Therefore, the maximum achievable strength is expected to be lower for carbon fibers compared to CNFs. This expectation is substantiated when comparing the theoretical limits of the strength of CNFs (as high as 14 GPa, see the following section), with intrinsic strength of CFs (at zero length), 6–10 GPa [1,34]. The theoretical strength of electrospun CNFs refers to a nanofiber composed almost entirely of turbostratic domains with their basal planes parallel to the nanofiber axis, while the intrinsic strength of CFs refers to a carbon fibers with a skin–core structure, with a core consisting of randomly oriented graphitic particles or amorphous carbon, without any defects such as voids, which could have evolved due to skin–core inhomogeneity.

### 2.5. Estimation of Upper Limit of the Intrinsic Strength of CNFs

Considering the radial inhomogeneity in PAN-based CFs as one of their inherent flaws which adversely affects their strength, the curious mind may consider the potentials of CNFs as a natural replacement for CFs, in which radial inhomogeneity can be eliminated by downsizing the technology of carbon fibers to submicron scales. Despite this potential for CNFs, their measured strength is in the range of ~0.8–8 GPa [3,15,21], which nearly overlaps with the corresponding values of carbon fibers, 2–6 GPa [13,28]. A comparison between the microstructure of carbon fibers and CNFs points to a lack of graphitic alignment in CNFs, as a major structural deficiency to be modified in order to improve the mechanical performance of CNFs [3,15,31]. However, it is to be emphasized that the lack of graphitic alignment is not inherent to CNFs [21]. For instance, inspired by carbon fiber technology, graphitic alignment in electrospun CNFs can be achieved by inducing molecular alignment in their precursors, as with hot drawing [25].

**Figure 2.**
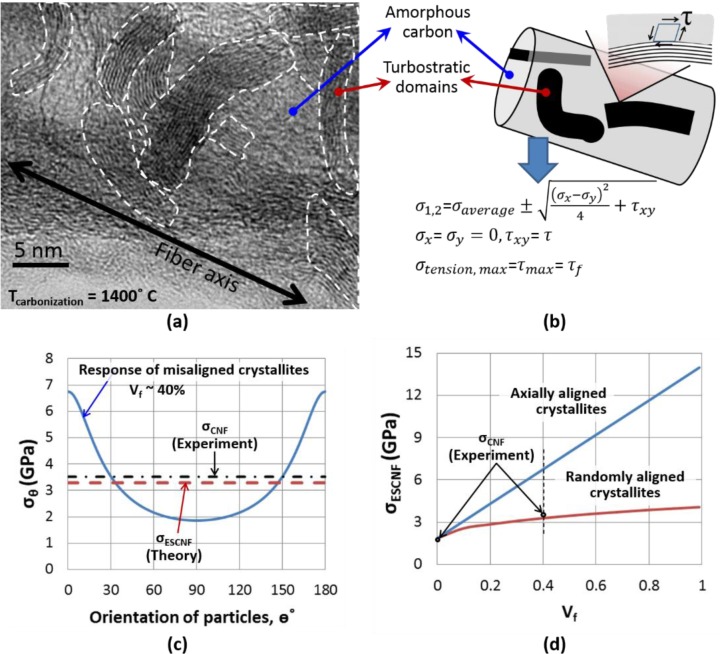
(**a**) TEM image of CNFs, with permission from [3], Copyright 2014 Elsevier Carbon; (**b**) modeled as amorphous carbon–matrix composites reinforced with turbostratic domains, encircled with broken lines in (**a**); (**c**) Strength of the composite is a function of the angle of the loading direction with respect to the basal plane of the crystallites; (**d**) Predictions of the model and corresponding experimental results [3] for the strength of the CNFs as a function of alignment of crystalline domains and degree of crystallinity.

Further insight into the mechanical properties of CNFs as a function of their microstructural parameters can be obtained through analytical models. To this end, we developed a continuum-based model in which each CNF is considered to be a composite material, composed of amorphous carbon (matrix), reinforced with turbostratic domains (reinforcements) in Figure 2a,b. Given the relatively low aspect ratio of reinforcements (turbostratic domains with a length of ~5–25 nm and a thickness of ~1–4 nm), the strength of individual CNFs can be predicted using Tsai–Hill criterion for the failure of short fiber composites [35]. In this model, the matrix is assumed to be isotropic, while the reinforcements demonstrate anisotropic mechanical behavior, with their strength being the highest when loaded parallel to the axis of the reinforcements (=σ*_f_*). According to the model, the strength of a portion of composite with unidirectional reinforcements, in which the direction of loading makes an angle θ with the axis of the reinforcement (the basal plane of turbostratic domains in our case), σ_(θ)_, is estimated as:

σ(θ)=[cos4θσL2+(1τf2−1σL2)cos2θsin2θ+sin4θσT2]−12with σL={Vfσf(1−lc2l)+(1−Vf)σml≥lCVfτf(ld)+(1−Vf)σml≤lCand lC=σf2τd (1)

where σ*_m_* is the strength of the matrix (amorphous carbon), σ*_f_* is the axial strength of the reinforcement (in plane strength of turbostratic domains), σ*_L_* and σ*_T_* are the strength of the composite parallel and perpendicular to reinforcement, respectively, τ*_f_* is shear strength of the interaction between the reinforcement and the matrix, *V_f_* is the volume fraction of reinforcements, *l* and *d* are length and diameter of the reinforcements, respectively, and *l_c_* is the critical length of the reinforcements. Reinforcements shorter than *l_c_* will be pulled out before composite failure; therefore, they will not experience mechanical failure. In this case, the strength of the composite does not depend on the strength of the reinforcement. Analogous to CNFs, the value of strength presented by Equation (1) represents the strength of a portion of the nanofiber in which the basal plane of the turbostratic domains make an angle θ with the axial direction of the fiber. Therefore, the strength of the CNF where turbostratic layers are randomly oriented, can be estimated as [35]:

$$\sigma_{\text{CNF}}\approx \frac{1}{\pi }\int_{0}^{\pi }\sigma_{\left(\theta \right)}\text{dθ}$$ (2)

This model is calibrated against the measured strength of individual CNFs with randomly oriented turbostratic domains [3]. In a study by PI and his colleagues, CNFs which were carbonized at 800 °C from the base material were mostly amorphous, with turbostratic particles thinner than 1 nm. Therefore, the strength of these CNFs (1.86 ± 0.55 GPa) is approximated to be equal to the strength of amorphous carbon (matrix in our model) obtained by carbonizing electrospun PAN nanofibers, σ*_m_*. Assuming that enough crosslinks exist between graphene sheets in the turbostratic domains, this strength is approximately equal to the transverse strength of the composite, σ*_T_*. In other words, for a turbostratic domain surrounded by amorphous carbon, sufficient loading in the direction normal to the basal plane is assumed to initiate failure in the amorphous carbon near the border with the turbostratic domain.

Another parameter required to predict the strength of CNFs is τ*_f_*, shear strength of the interaction between turbostratic domains and amorphous carbon. To estimate this parameter, we consider a hypothetical loading of the interface between turbostratic domains and amorphous carbon in pure shear (Figure 2b). This loading condition will induce principal tension and compression stresses with the same magnitude as the pure shear stress (a Mohr circle with the radius of the shear stress). The tensile stress is likely to induce failure in amorphous carbon due to its brittle nature, as evidenced by the conchoidal fracture surface of CNFs [15]. Hence, when loaded in pure shear, τ*_f_* is estimated to be equal, in magnitude, to the principal tensile stress of the matrix at failure, *i.e.*, τ*_f_* ≈ σ*_T_* = 1.86 GPa (Figure 2b).

The calibrated model presented in Equations (1) and (2) allows us to calculate the strength of CNFs at varying degrees of crystallinity (turbostratic domain concentration) and alignment. The accuracy of this model is checked by comparing the model predictions with the experimental results presented in [3]. As demonstrated by the PI and colleagues [3], increasing the carbonization temperature from 800 to 1400 °C, increases the turbostratic domain thickness from ~1 nm to ~3 nm. Based on TEM images of their CNFs, the average length and volume percentage of turbostratic layers at 1400 °C is estimated to be ~20 nm and 40%, respectively. The latter was estimated as the relative area of the turbostratic layers in the TEM images of CNFs. In these nanofibers, the maximum average stress in each turbostratic particle is thus estimated as

$$(2\pi r)\left(\frac{l}{2}\right)\frac{\tau_{f}}{\pi r^{2}}=\frac{\tau_{f}l}{r}\approx 14\text{ GPa}$$ (3)

This amount of stress in turbostratic layers is substantially lower than the strength of graphene sheets [5]. Therefore, the likely cause of the failure of CNFs is the detachment of the graphitic particles from amorphous carbon and pull-out. As a result, Equation (1), for the case of *l* < *l_c_*, in combination with Equation (2) can be used to estimate the strength of the CNFs. Based on this analysis, the strength of CNFs which were carbonized at 1400 °C are estimated to be ~3.29 GPa, in good agreement with the experimental measurements of 3.52 ± 0.64 GPa [3]. The high degree of crystallinity can be achieved by increasing the carbonization temperatures.

According to this preliminary analysis, high degrees of alignment of crystallites with the fiber axis (at θ ~ 0° in Figure 2c), without changing the degree of crystallinity, while maintaining *v_f_* ~ 40%, can increase strength of CNFs by ~100% to ~7 GPa (the vertical broken line in Figure 2d). In addition, with basal planes of the turbostratic domains oriented parallel with the nanofiber axis, the model predicts higher strengths with increased crystallinity (Figure 2d). According to the model, if CNFs are almost entirely composed of turbostratic domains with their basal planes parallel to nanofiber axis, an upper limit for the strength of CNFs, ~14 GPa, can be achieved (Figure 2d).

The comparison between the theoretical limits of the strength of CNFs (~14 GPa), and carbon fibers (as high as 7.5 GPa [28]) further points to the importance of fundamental research on CNFs. As stated previously, the theoretical strength of CNFs refers to a nanofiber composed almost entirely of turbostratic domains with their basal planes parallel to the nanofiber axis, while in carbon fibers the theoretical strength refers to carbon fibers with a skin–core structure without defects such as voids.

## 3. Concluding Remarks

The preceding sections discuss factors differentiating the morphology and potentially the mechanical properties of CFs from CNFs despite their similar processing steps. The defects caused by radial inhomogeneity, such as residual stresses and microcracks due to thermal expansion coefficient mismatch between the core and the skin, will reduce the strength of CFs, to the extent that the strength of CFs with aligned turbostratic domains on the skin is comparable to that of CNFs, which are mostly composed of randomly oriented turbostratic domains (~1–8 GPa). Moreover, our analytical modeling of CNFs, as composites of turbostratic domains in amorphous carbon, suggests that graphitic alignment in CNFs can be highly beneficial in enhancing their strength. In this regard, inspired by the technology of carbon fibers, hot drawing of PAN precursors may be considered as a scalable methodology. Hot drawing will align PAN molecules, leading to the alignment of ladder structure of stabilized PAN. The latter will transform into graphitic alignment in carbonized structures. Hot drawing can be carried out in water or air. Stretching in water/steam at temperatures close to boiling point of water can help to break the hydrogen bonds of PAN, and facilitate sliding between chains and fiber stretching [36]. Care must be taken when drawing in water as this can lead to pore formation in the fiber [37]. For stretching in air, higher temperatures are required, in the range of 130–180 °C, to generate sufficient chain mobility [38].

In case of PAN electrospun nanofiber bundles as precursors of CNF bundles, care should be taken to minimize the breakage of individual fibers during hot drawing [27] by, for example, employing sufficiently low rates of drawing to promote stress relaxation and delay strain localization [3]. Moreover, hot drawing and thermal stabilization of dense bundles of CNFs may lead to the development of cold junctions between CNFs [14], which may adversely affect the strength of CNFs, as junctions will act as stress concentration sites on CNFs. In addition, friction between individual fibers can cause breakage or local thinning of the fibers. Therefore, mechanical tests on bundles with low density and individual CNFs are expected to better represent the effect of hot drawing on mechanical strength of CNFs [3,15].

The future research on CNFs should also consider PAN copolymers and the inclusion of stabilization initiators such as phosphoric acid and nanoscale particles, such as CNTs, to enhance the quality of carbonization and graphitization [16,39,40]. With respect to nanoparticles, several aspects need to be considered, including the reinforcing effect of CNTs, stress localization and the graphitic templating effect. The first two are rooted in the significantly higher strength of CNTs compared to CNFs and elastic mismatch between them, respectively, while the last one is primarily the effect of the graphitic structure of CNTs. In addition, agglomeration, entanglement and uniform dispersion of CNTs remains a topic of concern creating hindrance in achieving the desired properties of the PAN/CNT fiber. Other catalyst particles have been also studied for improving graphitic alignment of the PAN derived CNF. Iron (III) acetylacetonate (AAI) has been used and has shown to improve the inplane size of graphite crystals [41]. Although these particles appear to improve graphitization of CNF fibers even at temperatures as low as 900–1300 °C, the graphitic crystals in the CNF form curved structures. As a result, it is questionable whether AAI can be used to improve overall alignment of graphitic planes in the CNF.

## Figures and Tables

**Table 1.** Diameters of electrospun CNFs and their skins (samples subjected to accelerated stabilization at 294 °C).

Sample #	Diameter of CNF (nm)	Hollow or filled	Skin thickness(nm)
1	133	Filled	–
2	209	Filled	–
3	375	Filled	–
4	428	Filled	–
5	584	Hollow	250
6	747	Hollow	308
7	829	Hollow	344
8	965	Hollow	369
